# 1 V Electronically Tunable Differential Difference Current Conveyors Using Multiple-Input Operational Transconductance Amplifiers

**DOI:** 10.3390/s24051558

**Published:** 2024-02-28

**Authors:** Montree Kumngern, Fabian Khateb, Tomasz Kulej, Lukas Langhammer

**Affiliations:** 1 Department of Telecommunications Engineering, School of Engineering, King Mongkut’s Institute of Technology Ladkrabang, Bangkok 10520, Thailand; montree.ku@kmitl.ac.th; 2 Department of Microelectronics, Brno University of Technology, Technická 10, 601 90 Brno, Czech Republic; 3 Faculty of Biomedical Engineering, Czech Technical University in Prague, nám. Sítná 3105, 272 01 Kladno, Czech Republic; 4 Department of Electrical Engineering, Brno University of Defence, Kounicova 65, 662 10 Brno, Czech Republic; lukas.langhammer@unob.cz; 5 Department of Electrical Engineering, Czestochowa University of Technology, 42-201 Czestochowa, Poland; kulej@el.pcz.czest.pl

**Keywords:** second-generation current conveyor (CCII), differential difference current conveyor (DDCC), operational transconductance amplifier (OTA), voltage-to-current converter, current-mode universal filter

## Abstract

This paper presents electronically tunable current conveyors using low-voltage, low-power, multiple-input operational transconductance amplifiers (MI-OTAs). The MI-OTA is realized using the multiple-input bulk-driven Metal Oxide Semiconductor transistor (MIBD-MOST) technique to achieve minimum power consumption. The MI-OTA also features high linearity, a wide input range, and a simple Complementary Metal Oxide Semiconductor (CMOS). Thus, high-performance electronically tunable current conveyors are obtained. With the MI-OTA-based current conveyor, both an electronically tunable differential difference current conveyor (EDDCC) and a second-generation electronically tunable current conveyor (ECCII) are available. Unlike the conventional differential difference current conveyor (DDCC) and second-generation current conveyor (CCII), the current gains of the EDDCC and ECCII can be controlled by adjusting the transconductance ratio of the current conveyors. The proposed EDDCC has been used to realize a voltage-to-current converter and current-mode universal filter to show the advantages of the current gain of the EDDCC. The proposed current conveyors and their applications are designed and simulated in the Cadence environment using 0.18 μm TSMC (Taiwan Semiconductor Manufacturing Company) CMOS technology. The proposed circuit uses ±0.5 V of power supply and consumes 90 μW of power. The simulation results are presented and confirm the functionality of the proposed circuit and the filter application. Furthermore, the experimental measurement of the EDDCC implemented in the form of a breadboard connection using a commercially available LM13700 device is presented.

## 1. Introduction

In the last decade, more and more attention has been paid to the current-mode technique in electronic circuit design. This technique can offer advantages in certain applications in terms of high-speed operation, bandwidth, accuracy, and simplified signal processing. Arithmetic operations, such as the addition, subtraction, and multiplication of signals in current forms are simpler compared to voltage-mode circuits. In other words, the addition and subtraction of signals in voltage forms based on operational amplifiers (op-amps) suffer from many passive resistors [1]. Moreover, current-mode circuits can be designed almost exclusively using current-mode devices because they do not need high current gain or high-precision passive elements. For example, an op-amp-based inverting amplifier offers a high-precision transfer function when there is high voltage gain (infinite for the ideal case) and high-precision passive elements are available [1].

Second-generation current conveyors (CCII) [2] are well-known active devices for realizing current-mode circuits, such as current-mode filters [3,4,5,6], current-mode oscillators [7,8,9,10], and current-mode rectifiers [11,12,13]. Furthermore, there are several current conveyors available according to the open literature, such as differential difference current conveyors (DDCC) [14], differential voltage current conveyors (DVCC) [15], fully differential current conveyors (FDCCII) [16], and fully balanced second-generation current conveyors (FBCCII) [17]. These current conveyors [14,15,16,17] are designed to enhance performance in terms of holding the input signals and/or output signals in differential forms.

Nowadays, CMOS active devices operating with low supply voltage and power consumption are of interest because they are required for applications in portable electronics, sensors, and biomedical systems. Power consumption is also a key parameter for researchers in the design of conventional electronic circuits. Focusing on current conveyors, low-voltage and low-power current conveyors are available according to the open literature, i.e., CCII in [18,19,20], DDCC in [21,22], FDCCII in [23,24,25], and FBCCII in [26,27,28].

A conventional CCII usually has three terminals (x-, y-, and z-terminals) [2], and its terminal relationships are v_y_ = v_x_ and i_z_ = i_x_. It should be noted that the voltage and current gains of a conventional CCII are equal to one. To increase the functionality of the CCII by offering electronic tuning of the current gain between the x- and z-terminals, electronically tunable CCIIs (ECCIIs) have been proposed [29,30,31,32,33,34,35,36]. In [29], the ECCII was first designed using an op-amp and an operational transconductance amplifier (OTA). ECCIIs can also be implemented using bipolar technology [30,31] and CMOS technology [32,33,34]. An electronically tunable differential difference current conveyor (EDDCC) was also proposed in [35,36]. The ECCIIs and EDDCCs are used as the basic building blocks of universal filters [37,38,39,40] and oscillators [41,42,43,44,45,46,47]. The current gain of the current conveyors can be used as a design parameter for applications, such as tuning the quality factor of the filters [37,38,39], adjusting the current gain of filter functions [40], selecting a single circuit to operate as either a filter or an oscillator [41], and controlling the condition of oscillation and/or the oscillator’s frequency of oscillation [42,43,44,45,46,47]. It should be noted that the ECCII and EDDCC in [29,30,31,32,33,34,35,36] do not provide low-voltage and low-power operations, i.e., ±1.5 V of supply voltage [33], ±2.5 V of supply voltage [34,35], and ±5 V of supply voltage [31,36]. Although the current conveyors in [18,19,20,21,22,23,24,25,26,27,28] provide low-voltage and low-power operations, the current gain between the x- and z-terminals of these current conveyors is not provided.

Therefore, this paper presents low-voltage low-power current conveyors that offer current gain between the x- and z-terminals. The electronically tunable current conveyors have been designed using low-voltage, low-power, multiple-input OTAs (MI-OTAs). The current gain of the proposed electronically tunable current conveyor can be controlled by adjusting the ratio of transconductances of the current conveyors. The MI-OTA is realized using the multiple-input bulk-driven MOS transistor (MIBD-MOST) technique to obtain minimum voltage supply and power consumption [48]. Recently, multiple-input OTAs have been utilized in many interesting applications that exhibit a minimal number of active elements, power supply, and reduced complexity [48,49]. By using a MI-OTA-based electronically tunable current conveyor, we can obtain an electronically tunable differential difference current conveyor (EDDCC) and an electronically tunable second-generation current conveyor (ECCII). The EDDCC has been used to realize the voltage-to-current (V-to-I) converter and current-mode universal filter. The performances of the proposed current conveyors and their applications were evaluated in the Cadence environment using 0.18 μm CMOS technology from TSMC. The proposed current conveyors use ±0.5 V of power supply and consume 90 μW of power. The EDDCC has also been implemented in the form of a breadboard connection in order to perform experimental measurements. The proposed EDDCC can be used for voltage- and current-mode sensor applications or as a conditioning circuit for processing biological signals that require low supply voltages and reduced power consumption.

The paper is organized as follows: Section 2 describes the structure of the MI-OTA and the proposed EDDCC. The applications of the EDDCC as a V-to-I converter and current-mode universal filter are shown in Section 3. The simulation results of the proposed ECCII, the V-to-I converter, and the universal filter are shown in Section 4. Section 5 describes the experimental measurement results of the EDDCC. Finally, Section 6 concludes the paper.

## 2. Proposed Electronically Tunable Current Conveyors

### 2.1. The Multiple-Input Operational Transconductance Amplifier

**Figure 1:**
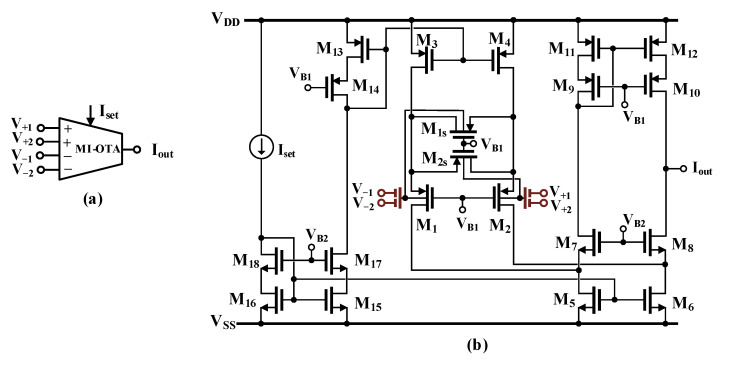
MI-OTA (**a**) symbol and (**b**) realization using the MIBD-MOST technique.

The symbol and the CMOS realization of the multiple-input OTA proposed in this work are shown in Figure 1a and 1b, respectively. The output current *I_out_* can be described by the following equation:

(1) $$I_{out}=g_{m}\left(V_{+1}+V_{+2}-V_{-1}+V_{-2}\right)$$

where *g_m_* is the small-signal transconductance.

Overall, the circuit can be considered as a folded cascode OTA, with the input bulk-driven differential pair M_1_, M_2_ linearized using the triode region transistors M_1s_ and M_2s_. A similar linearization technique was proposed by Krummenacher and Joehl [50] for a gate-driven transconductor operating in the strong inversion region. Figure 1 presents a BD counterpart of the circuit, operating in weak inversion, and with the input transistors replaced by multiple-input devices. Such a version of the input stage was first proposed and verified experimentally in [49].

**Figure 2:**
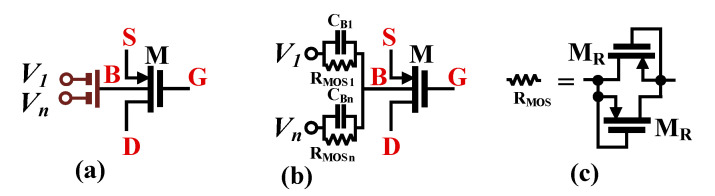
MIBD-MOST technique (**a**) symbol, (**b**) realization, and (**c**) R_MOS_ realization.

The practical realization of multiple-input devices is shown in Figure 2. Note that multiple inputs were realized using a capacitive voltage divider/analog summer, composed of the capacitors *C_Bi_*. The large resistors *R_MOSi_*, connected in parallel to the capacitors, are used to properly bias the bulk terminal of the transistor for DC. They are realized as an anti-parallel connection of two minimum-size MOS transistors operating in the cut-off region. Due to their high resistances, their impact on the voltage transfer function of the input divider can be neglected for working frequencies of *ω* > 1/*C_Bi_R_MOSi_*. In such a case, the AC voltage at the bulk terminal of the device can be expressed as follows:

(2) $$V_{b}=\sum_{i=1}^{n}\beta_{i}V_{i}$$

where n is the number of inputs and *β_i_* is the voltage gain of the input capacitive divider from *i*th input. Neglecting second-order effects, *β_i_* can be expressed as follows:

(3) $$\beta_{i}=\frac{C_{Bi}}{\sum_{i=1}^{n}C_{Bi}}$$

Note that with identical *C_Bi_*, *β_i_* = 1/*n* for *i* = 1, …, *n*.

The MOS transistor with a capacitive input divider can be seen as a new active device called a bulk-driven multiple-input MOS transistor [48]. The use of such devices enables the realization of input signal summation (see Equation (1)) without the need for a second input stage, thus simplifying the overall structure and saving dissipated power.

Regarding the input stage of the OTA, its linearity depends on the parameter *k*, which is defined as follows:

(4) $$k=\frac{{\left(W/L\right)}_{1s,2s}}{{\left(W/L\right)}_{1,2}}$$

The best linearity of the input stage is achieved for *k* = 0.5 [49], i.e., the same value as for its GD counterpart operating in weak inversion [51]. This result does not depend on the value of the biasing current I_set_ if the operation in weak inversion is provided.

The rest of the OTA structure is rather conventional, with its cascode output stage M_5_-M_12_. The transistors M_13_-M_18_ are used for biasing purposes. All the transistors in the OTA circuit, except M_1s_ and M_2s_, should operate in the penthode region.

The small-signal transconductance of the OTA can be expressed as follows [49]:

(5) $$g_{m}=\beta \cdot \eta \frac{4k}{4k+1}\cdot \frac{I_{set}}{n_{p}U_{T}}$$

where *η* = *g_mb_*_1,2_/*g_m_*_1,2_ is the bulk-to-gate transconductance ratio of the input pair at the operating point, n_p_ is the subthreshold slope factor for p-channel devices, *U_T_* is the thermal potential, and the other symbols are explained earlier.

As can be concluded from (5), the resulting transconductance is attenuated by the input capacitive divider and by the application of bulk-driven devices (note that both the capacitive divider gain *β* and the bulk-to-gate transconductance ratio *η* are less than unity). The transconductance is proportional to the biasing current I_set_, and thus can be linearly regulated by this current.

The relatively low value of the overall transconductance also decreases the voltage gain of the OTA. However, thanks to the high-resistance cascode output stage, the DC voltage gain of the OTA is maintained at a sufficient level as follows:

(6) $$A_{V}≅g_{m}[\left(g_{m8}r_{ds8}r_{ds6}\right)||\left(g_{m10}r_{ds10}r_{ds12}\right)]$$

The input capacitive divider, as well as the bulk-driven technique, extend the linear range of the OTA 1/(*βη*) times. However, the input-referred noise is increased in the same proportion; thus, the dynamic range of the circuit remains unchanged as compared to its GD counterpart. Nevertheless, application of the bulk-driven technique, combined with an additional capacitive divider, simplifies the design of analog blocks in an ultra-low-voltage environment and avoids hard nonlinearities for a relatively large input voltage swing.

### 2.2. Proposed Electronically Tunable Current Conveyors

**Figure 3:**
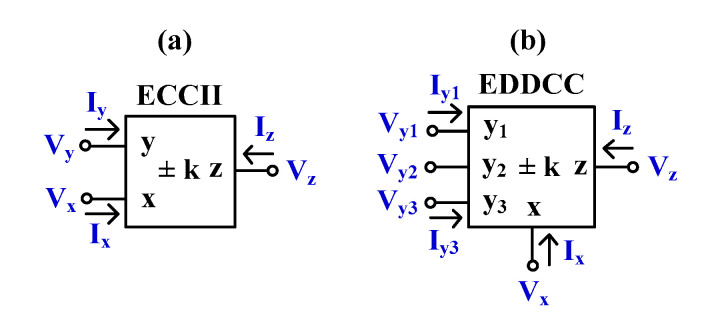
The symbol of electronically tunable current conveyors (**a**) ECCII and (**b**) EDDCC.

Figure 3a shows the symbol of the electronically tunable second-generation current conveyor (ECCII) and Figure 3b shows the electrical symbol of the electronically tunable differential difference current conveyor (EDDCC). The port characteristics of the ECCII and EDDCC can be expressed, respectively, as follows:

(7) $$\left(\begin{array}{c} I_{y}\\ V_{x}\\ I_{z} \end{array}\right)=\left(\begin{array}{ccc} 0 & 0 & 0\\ 1 & 0 & 0\\ 0 & \pm k & 0 \end{array}\right)\left(\begin{array}{c} V_{y}\\ I_{x}\\ V_{z} \end{array}\right)$$

(8) $$\left(\begin{array}{c} I_{y1}\\ I_{y2}\\ I_{y3}\\ V_{x}\\ I_{z} \end{array}\right)=\left(\begin{array}{ccccc} 0 & 0 & 0 & 0 & 0\\ 0 & 0 & 0 & 0 & 0\\ 0 & 0 & 0 & 0 & 0\\ 1 & -1 & 1 & 0 & 0\\ 0 & 0 & 0 & \pm k & 0 \end{array}\right)\left(\begin{array}{c} V_{y1}\\ V_{y2}\\ V_{y3}\\ I_{x}\\ V_{z} \end{array}\right)$$

The characteristics of the ECCII and EDDCC are similar to the conventional CCII [2] and DDCC [14], except for the current gain between the x- and z-terminals, which can be given by *k*.

**Figure 4:**
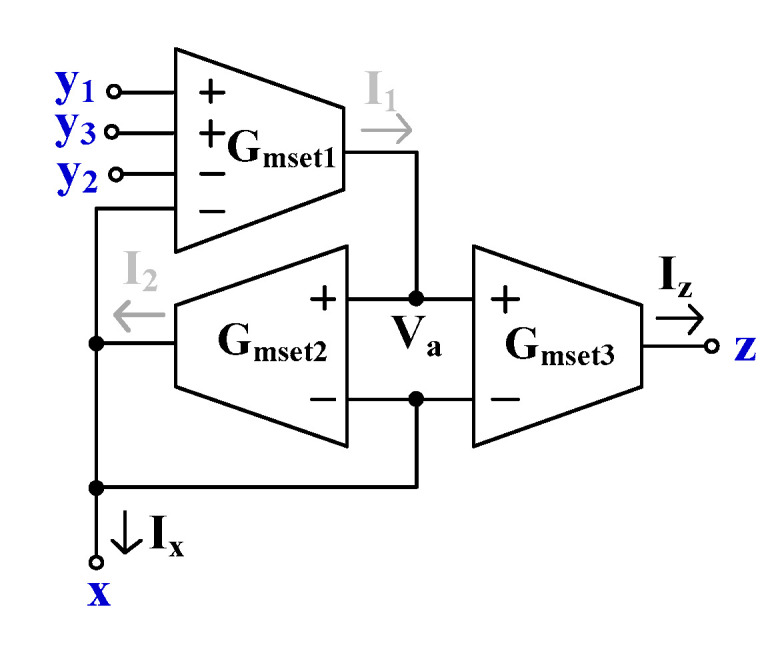
Proposed EDDCC using MI-OTAs.

Figure 4 shows the proposed EDDCC using MI-OTAs. This circuit can also work as an ECCII if the y_1_-terminal is the input and the y_2_- and y_3_-terminals are connected to ground. It can further work as an inverting ECCII if the y_2_-terminal is the input and the y_1_- and y_3_-terminals are connected to ground.

To explain the operation of the proposed EDDCC, it is assumed that all OTAs are identical. Using (1), the currents *I*_1_, *I*_2_, and *I*_3_ in Figure 4 can be expressed as follows:

(9) $$I_{1}=G_{mset1}\left(V_{y1}-V_{y2}+V_{y3}-V_{x}\right)$$

(10) $$I_{x}=G_{mset2}\left(V_{a}-V_{x}\right)$$

(11) $$I_{z}=G_{mset3}\left(V_{a}-V_{x}\right)$$

The OTA_2_ of *G_mset_*_2_ is connected as a negative-feedback-like voltage follower (VF) circuit. Thus, *V_a_* = *V_x_* and this voltage (i.e., *V_a_* = *V_x_*) is fed to the inverting input terminal of OTA_1_ (*G_mset_*_1_). Therefore, this OTA_1_ is also operated as a VF. The voltage relationship of the EDDCC in Figure 4 can be given as follows:

(12) $$V_{x}=V_{y1}-V_{y2}+V_{y3}$$

The addition and subtraction voltage properties of the EDDCC can be obtained.

By substituting (10) into (11), the relationship of the currents $I_{x}$ and $I_{z}$ can be expressed as follows:

(13) $$I_{z}=\left(\frac{G_{mset3}}{G_{mset2}}\right)I_{x}$$

(14) $$k=\frac{G_{mset3}}{G_{mset2}}$$

Thus, the current gain of EDDCC can be varied by adjusting the ratio of *G_mset_*_3_/*G_mset_*_2_ (*G_mset_*_3_/*G_mset_*_2_ = *k*).

## 3. Applications of the EDDCC

### 3.1. V-to-I Converter Using EDDCC

**Figure 5:**
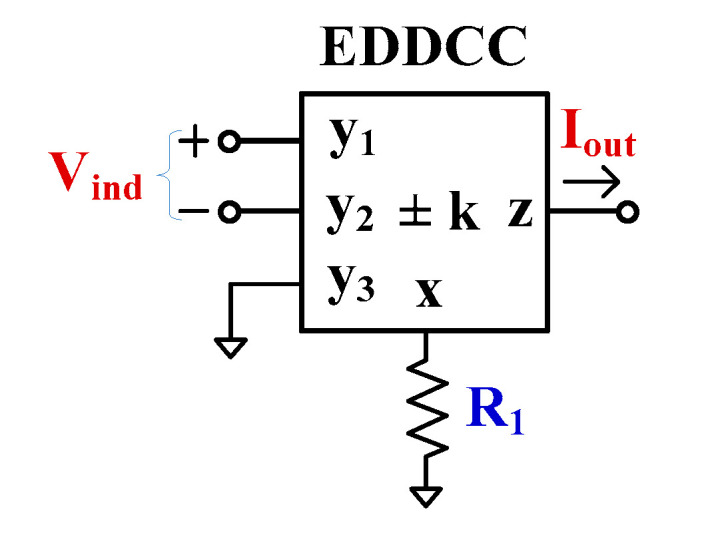
Applications of the EDDCC to V-to-I converter.

Voltage-to-current (V-to-I) converters, the so-called transconductors, are useful basic building blocks for realizing analog filters, oscillators, gyrators, and instrumentation amplifiers; for examples see [52,53,54,55,56]. In this work, the proposed EDDCC has been used to realize the V-to-I converter as shown in Figure 5. The voltage input ($V_{ind}=V_{in+}-V_{in-}$) is converted to the output current ($I_{out}$) by *R*_1_ and the current gain can also be adjusted by the current gain k of the EDDCC. The circuit can work as a single-ended V-to-I converter (non-inverting or inverting input) and a differential V-to-I converter. Using (8), the output current of the circuit in Figure 5 can be expressed as follows:

(15) $$I_{out}=k\left(\frac{1}{R_{1}}\right)V_{ind}$$

where $k=G_{mset3}/G_{mset2}$ and $V_{ind}=V_{in+}-V_{in-}$.

### 3.2. Current-Mode Universal Filter Using EDDCCs

**Figure 6:**
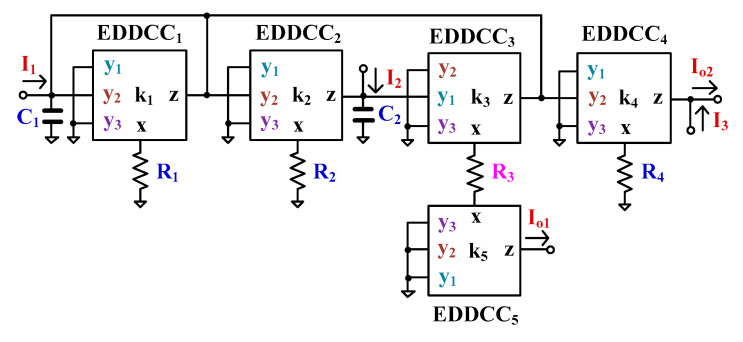
The proposed current-mode universal filter.

To show the advantages of the current gain of the EDDCC, the EDDCC has been used to realize a current-mode universal filter as shown in Figure 6. The filter employs five EDDCCs, four resistors, and two capacitors. The output terminals possess a high impedance level, and the circuit uses grounded capacitors, which is convenient for the implementation of integrated circuits. The filtering functions can be achieved through the appropriate use of input signals and appropriate selection of output signals.

Using (8) and nodal analysis, the output currents I_o1_ and I_o2_ can be expressed as follows:

(16) $$I_{o1}=k_{5}\frac{-k_{2}k_{3}I_{1}+\left(k_{3}sC_{1}R_{T}+k_{1}k_{3}\right)I_{2}}{s^{2}C_{1}C_{2}{R_{T}R}_{3}+k_{1}sC_{2}R_{3}+k_{2}k_{3}}$$

(17) $$I_{o2}=k_{4}\frac{k_{2}sC_{2}R_{3}I_{1}+k_{2}k_{3}I_{2}}{s^{2}C_{1}C_{2}R_{T}R_{3}+k_{1}sC_{2}R_{3}+k_{2}k_{3}}-I_{3}$$

where $R_{T}=R_{1}=R_{2}=R_{4}$.

The variants of the current-mode universal filter’s filtering functions are shown in Table 1. The proposed filter offers five standard filtering functions. Moreover, the current gains of LP and BP filters can be adjusted by *k*_4_ and *k*_5_ of EDDCC_4_ and EDDCC_5_.

The natural frequency ($\omega_{o}$) and quality factor (Q) can be expressed as follows:

(18) $$\omega_{o}=\sqrt{\frac{k_{2}k_{3}}{C_{1}C_{2}{R_{T}R}_{3}}}$$

(19) $$Q=\frac{1}{k_{1}}\sqrt{\frac{k_{2}k_{3}C_{1}R_{T}}{C_{2}R_{3}}}$$

The natural frequency can be given by *R_T_* and *R*_3_ (i.e., *R_T_* = *R*_3_) and the quality factor can be controlled independently and electronically by *k*_1_ of EDDCC_1_. The current gains of LP outputs *I_o_*_1_ and *I_o_*_2_ can be controlled by *k*_5_ and *k*_4_, respectively. In the case of tuning *Q* of the BP, the current gain will be equal to 1 if *k*_1_ = *k*_4_ for output *I*_o2_ or *k*_1_ = *k*_5_ for output *I*_o1_. The current gain of BP can be obtained if *k*_4_ > *k*_1_ (or *k*_5_ > *k*_1_).

### 3.3. Non-Ideal Analysis

Taking into account the non-idealities of the EDDCC, the relationship of the terminal voltages and currents can be rewritten as follows:

(20) $$\left(\begin{array}{c} I_{y1}\\ I_{y2}\\ I_{y3}\\ V_{x}\\ I_{z} \end{array}\right)=\left(\begin{array}{ccccc} 0 & 0 & 0 & 0 & 0\\ 0 & 0 & 0 & 0 & 0\\ 0 & 0 & 0 & 0 & 0\\ \alpha_{j1} & -\alpha_{j2} & \alpha_{j3} & 0 & 0\\ 0 & 0 & 0 & \pm \beta_{j}k_{j} & 0 \end{array}\right)\left(\begin{array}{c} V_{y1}\\ V_{y2}\\ V_{y3}\\ I_{x}\\ V_{z} \end{array}\right)$$

where *α_j_*_1_ = 1 − *ε_j_*_1*v*_ and *ε_j_*_1*v*_ (|ε_k1v_| « 1) denotes the voltage-tracking error from *V_y_*_1_ to *V_x_* of the *j*th EDDCC, *α_j_*_2_ = 1 − *ε_j_*_2*v*_ and *ε_j_*_2*v*_ (|*ε_j_*_2*v*_| « 1) denotes the voltage-tracking error from *V_y_*_2_ to *V*_x_ of the *j*th EDDCC, *α_j_*_3_ = 1 − ε_j3v_ and ε_j3v_ (|ε_j3v_| « 1) denotes the voltage-tracking error from V_y3_ to V_x_ of the *j*th EDDCC, and *β_j_* = 1 − *ε_i_* and *ε_i_* (*ε_i_* « 1) denotes the output current-tracking error of the *j*th EDDCC.

Using (20), the denominator of the proposed filter becomes as follows:

(21) $$s^{2}C_{1}C_{2}R_{T}R_{3}+k_{1}sC_{2}R_{3}\beta_{1}+k_{2}k_{3}\beta_{2}\beta_{3}\alpha_{12}\alpha_{21}\alpha_{31}$$

The natural frequency and quality factor become as follows:

(22) $$\omega_{on}=\sqrt{\frac{k_{2}k_{3}\beta_{2}\beta_{3}\alpha_{12}\alpha_{21}\alpha_{31}}{C_{1}C_{2}{R_{T}R}_{3}}}$$

(23) $$Q_{n}=\frac{1}{\beta_{1}k_{1}}\sqrt{\frac{k_{2}k_{3}C_{1}R_{T}\beta_{2}\beta_{3}\alpha_{12}\alpha_{21}\alpha_{31}}{C_{2}R_{3}}}$$

It follows from (22) and (23) that tracking errors change the natural frequency and the quality factor. However, it should be noted that the natural frequency can be easily compensated by adjusting k_2_ and k_3_ and the quality factor can be compensated by adjusting k_1_.

With respect to the parasitic parameters of the EDDCC on the current-mode universal filter, the parasitic impedances *R_z_* and *C_z_* at the z-terminal [5] are considered. From Figure 6, it can be seen that capacitor C_1_ is in parallel with parasitic capacitances *C_z1_*, *C_z3_* and parasitic resistances *R_z_*_1_, *R_z_*_3_ while capacitor *C*_2_ is in parallel with parasitic capacitance *C_z_*_2_ and parasitic resistances *R_z_*_2_. The parasitic effects on the pole frequency of the filter can be avoided by choosing *C*_1_ ≫ *C_z_*_1_ + *C_z_*_3_, *C*_2_ ≫ *C_z_*_2_, *R*_1_ ≪ *R_z_*_1_//*R_z_*_3_, and *R*_2_ ≪ *R_z_*_2_.

## 4. Simulation Results

The proposed EDDCC and its applications were simulated in the Cadence Virtuoso System Design Platform using 0.18µm CMOS technology from TSMC (Taiwan Semiconductor Manufacturing Company, Hsinchu Science Park, Taiwan). The aspect ratios of all MOS transistors of the MI-OTA in Figure 1 are listed in Table 2. The initial values of I_set1_ = I_set2_ = 5 μA, while the values of I_set3_ were changed to adjust the current gain k of the EDDCC.

**Figure 7:**
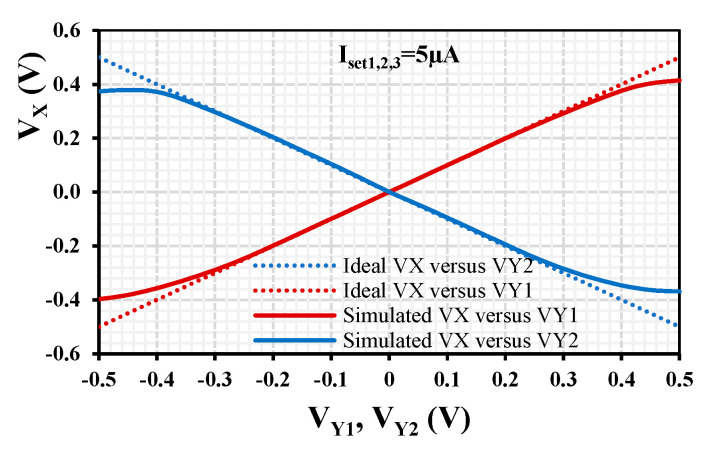
DC curves V_x_ versus V_y1_ (V_y2_ and V_y3_ are grounded) and V_x_ versus V_y2_ (V_y1_ and V_y3_ are grounded) showing the ideal and simulated input voltage range.

**Figure 8:**
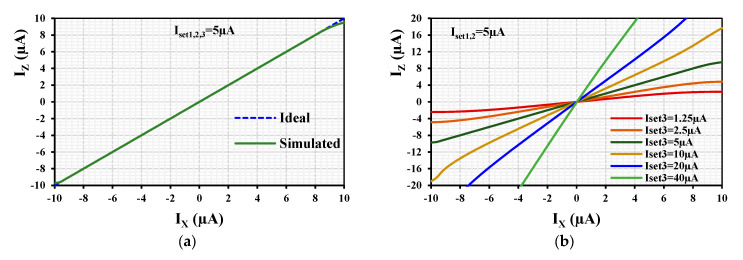
DC curves I_z_ versus I_x_ (**a**) with k = 1 and (**b**) with different values of k.

**Figure 9:**
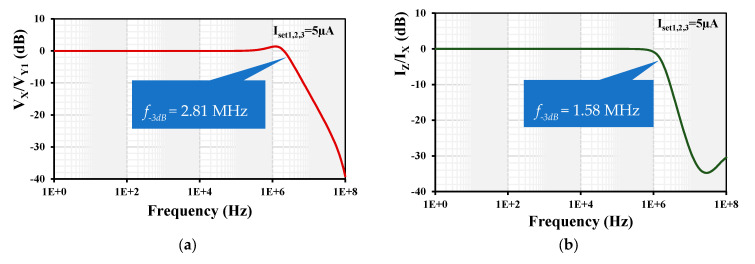
Frequency responses of (**a**) voltage gain V_x_/V_y1_ and (**b**) current gain I_z_/I_x_.

**Figure 10:**
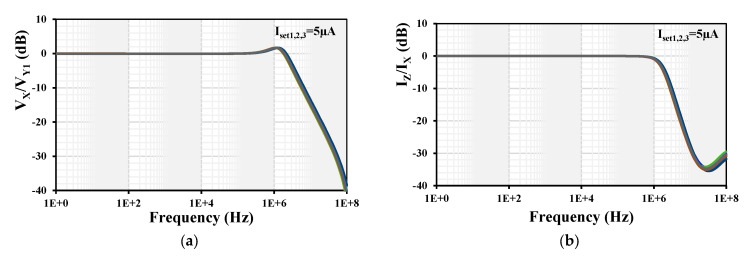
Frequency responses of (**a**) voltage gain V_x_/V_y1_ and (**b**) current gain I_z_/I_x_ with PVT analysis.

**Figure 11:**
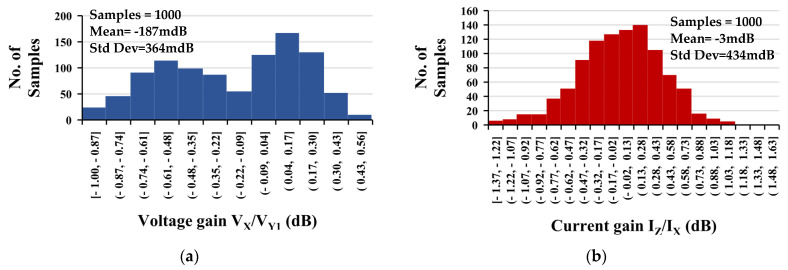
The histogram of the low-frequency (**a**) voltage gain V_x_/V_y1_ and (**b**) current gain I_z_/I_x_ with 1000 runs MC analysis.

**Figure 12:**
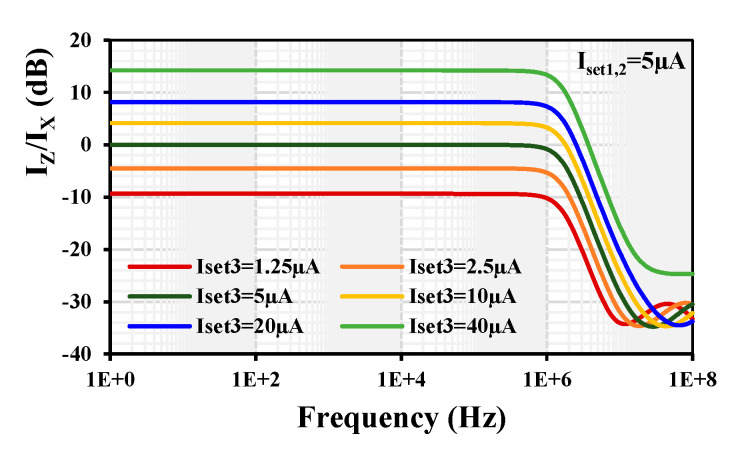
Frequency response of the current gain I_z_/I_x_ with different gain k.

For the EDDCC in Figure 3b, the supply voltage was chosen to be V_DD_ = –V_SS_ = 0.5 V, with the setting currents I_set1_ = I_set2_ = I_set3_ = 5 μA. The power consumption of the EDDCC was 90 μW. Figure 7, Figure 8, Figure 9, Figure 10, Figure 11 and Figure 12 show the simulation results of the EDDCC. Figure 7 shows the ideal and simulated DC voltage characteristics V_x_ versus V_y1_ (V_y2_ and V_y3_ are grounded) and V_x_ versus V_y2_ (V_y1_ and V_y3_ are grounded) when V_y1_ and V_y2_ were swept from −0.5 V to 0.5 V. A good linearity is evident for V_X_/V_Y1_ and V_X_/V_Y2_ with the input voltage range ±0.3 V.

Figure 8a shows the ideal and simulated DC current characteristics I_z_ versus I_x_ (with k = 1) when I_X_ was swept from −10 µA to +10 µA. The curves overlap in the range of ±9 µA. Figure 8b shows the I_z_ versus I_x_ for different k with a constant I_set1,2_= 5 µA and varied I_set3_= (1.25, 2.5, 5, 10, 20, 40) µA. The wide turnability of I_z_ versus I_x_ is evident.

The simulated frequency responses of the voltage gain V_x_/V_y1_ and the current gain I_z_/I_x_ are shown in Figure 9. The −3 dB bandwidths were 2.81 MHz and 1.58 MHz, and the low-frequency gains were −33.6 mdB and −76 µdB for the voltage V_x_/V_y1_ and current I_z_/I_x_ gains, respectively. It is worth noting here that a compensation capacitor of 2 pF was connected between the input of G_mset2_ to obtain a flat magnitude response of the current gain. Without this compensation capacitor, the peak is around 6 dB.

Process, voltage, temperature (PVT) corners were used to confirm the robustness of the design. The process transistor corners were fast-fast, fast-slow, slow-fast, and slow-slow; the process MIM capacitor corners were fast-fast and slow-slow; the voltage supply corners were = ±10% (V_DD_-V_SS_); and the temperature corners were −20 °C and 60 °C. The results for the frequency responses of the voltage gain V_x_/V_y1_ and current gain I_z_/I_x_ are shown in Figure 10. The −3 dB bandwidths were in range of (2.66 to 3) MHz and (1.49 to 1.72) MHz, and the low-frequency gains were in range of (−62.8 to 21.2) mdB and (−124.6 to 79.9) µdB for the voltage V_x_/V_y1_ and current I_z_/I_x_ gains, respectively. As is evident, the variations are within the acceptable range.

Monte Carlo (MC) analysis was used to perform the statistical analysis to estimate parametric yield and generate information about the performance characteristic of the frequency voltage gain V_x_/V_y1_ and current gain I_z_/I_x_ of the EDDCC. Figure 11 shows the histogram of a 1000 run MC analysis, showing the mean value to be −187 mdB and −3 mdB, and the standard deviation to be 364 mdB and 434 mdB for the voltage and current gains, respectively.

The simulated frequency response of the current gain I_z_/I_x_ with a constant I_set1,2_ = 5 µA and varied I_set3_= (1.25, 2.5, 5, 10, 20, 40) µA is shown in Figure 12. The simulated current gain k was varied to (−9.3, −4.48, 0, 4.13, 8.1, 14.22) dB, respectively. This result confirms that the proposed EDDCC can provide the current gain I_z_/I_x_.

**Figure 13:**
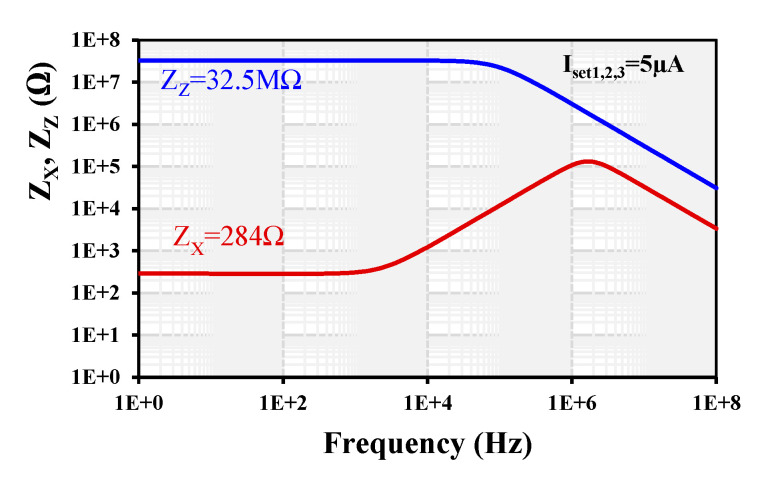
Frequency dependence of the parasitic impedances of x- and z-terminals.

The simulated frequency dependence of the parasitic impedances of the z- and x-terminals is shown in Figure 13. The resistance of the z-terminal is 32.5 MΩ and the resistance of the x-terminal is 284 Ω for I_set1,2,3_ = 5 µA.

**Figure 14:**
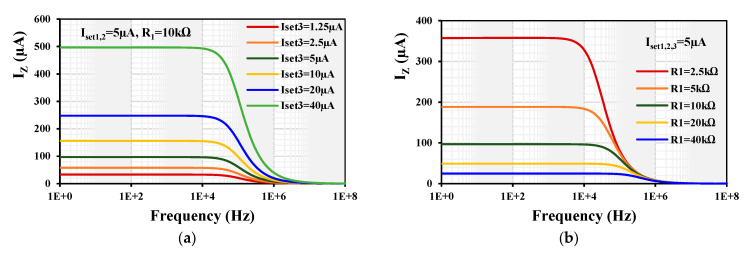
Frequency responses of the V-to-I converter against the current gain k for (**a**) R_1_ = 10 kΩ, I_set1,2_ = 5 µA, and various I_set3_= (1.25, 2.5, 5, 10, 20, 40) µA and (**b**) for I_set1,2,3_ = 5 µA and various R_1_ = (2.5, 5, 10, 20, 40) kΩ.

Figure 14a shows the frequency responses of the V-to-I converter shown in Figure 5 against the current gain k for a constant R_1_ = 10 kΩ, I_set1,2_ = 5 µA, and various I_set3_= (1.25, 2.5, 5, 10, 20, 40) µA, and Figure 14b for I_set1,2,3_ = 5 µA and various R_1_ = (2.5, 5, 10, 20, 40) kΩ. The wide tunability of the current gain is evident.

**Figure 15:**
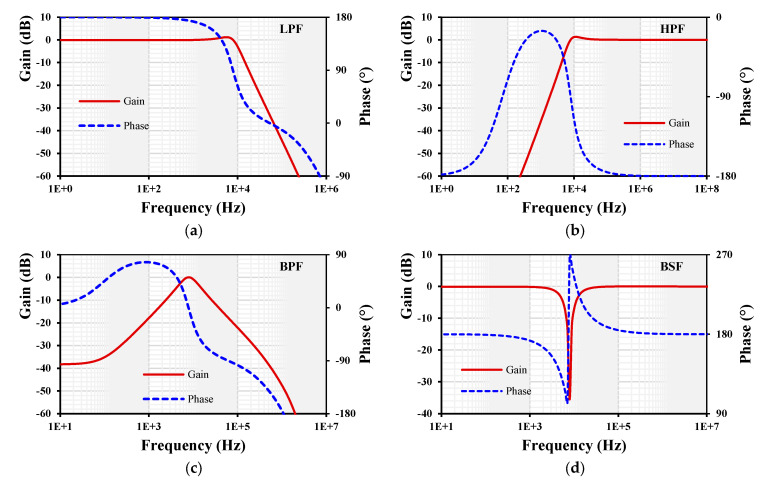

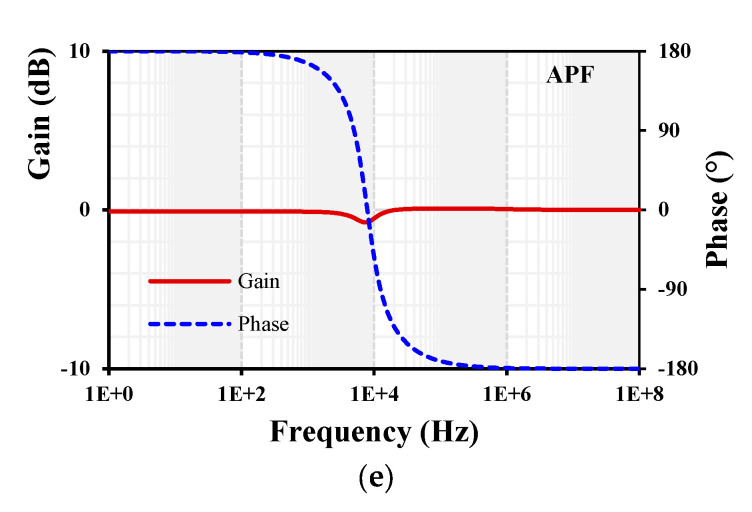
Gain and phase frequency responses of the current-mode filter: (**a**) LPF, (**b**) HPF, (**c**) BPF, (**d**) BSF, and (**e**) APF.

For simulation of the current-mode universal filter shown in Figure 6, the parameters C_1_ = C_2_ = 100 pF and R_1–4_ = 200 kΩ were chosen. The gain k_1–5_ = 1 was set by choosing the setting current of all EDDCC_1–5_ to be I_set_ = 5 µA. However, for the APF, the current of the EDDCC_4_ was set to I_set3_ = 13 µA in order to obtain k = 2. The gain and phase frequency characteristics are shown in Figure 15. The cut-off frequency was 7.9 kHz.

**Figure 16:**
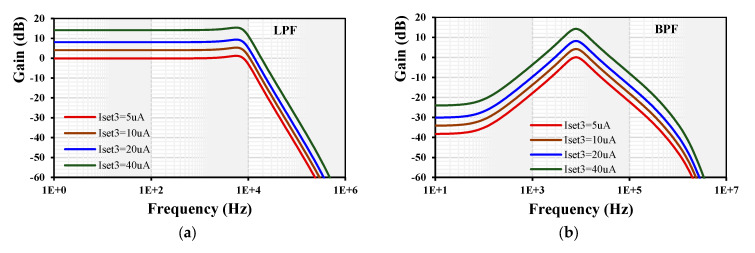
Gain and phase frequency responses of the current-mode filter: (**a**) LPF and (**b**) BPF.

Figure 16 shows the tuning capability of the gain for the LPF and BPF. The setting current of all EDDCC_1-4_ was set to be I_set_ = 5 µA while the k of EDDCC_5_ was changed by its I_set3_ = (5, 10, 20, 40) µA. The low-frequency gain of the LPF was around (0.1, 4, 8, 14) dB and for BPF, it was (0.02, 4.1, 8.1, 14.2) dB.

**Figure 17:**
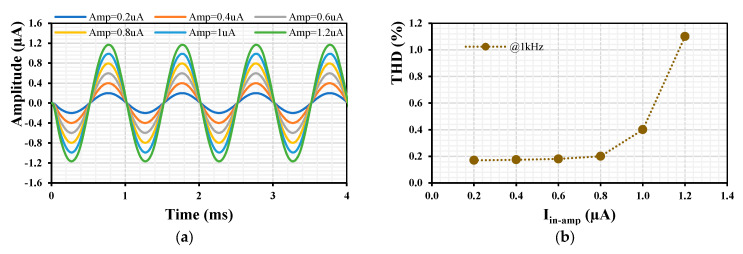
Transient response (**a**) and THD (**b**) of the LPF with different input signals and k = 1.

Figure 17 shows the transient response of the LPF’s output (a) and the total harmonic distortion (b) when an input signal at 1 kHz and different amplitudes (0.2, 0.4, 0.6, 0.8, 1, 1.2) µA were applied to the input of the filter. The gain was set to be k = 1. The THD of the output signal was below 1.2% for an input amplitude of 1.2 µA.

**Figure 18:**
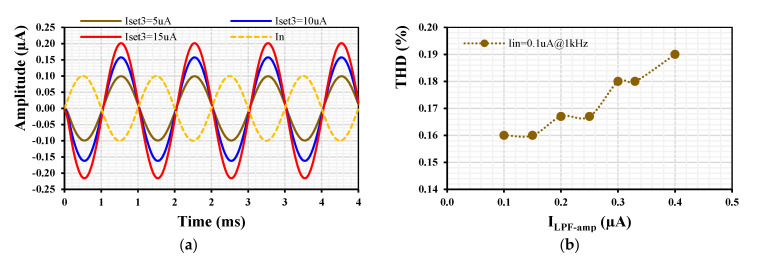
Transient response (**a**) and THD (**b**) of the LPF with a 0.1 µA @1 kHz input signal and various k_5_.

Figure 18a shows the transient response of the LPF when a sine wave I_in_ = 0.1 µA@1kHz is applied to the input of the filter with k_1-4_ = 1 (I_set_ = 5 µA) while the gain k_5_ of the EDDCC_5_ is varied by its I_set3_ = (0.5, 10, 15) µA. The output signal of the LPF is inverted and amplified as expected. The THD is shown in Figure 18b, where the 0.19% THD is shown for a 0.4 µA amplitude output signal.

**Figure 19:**
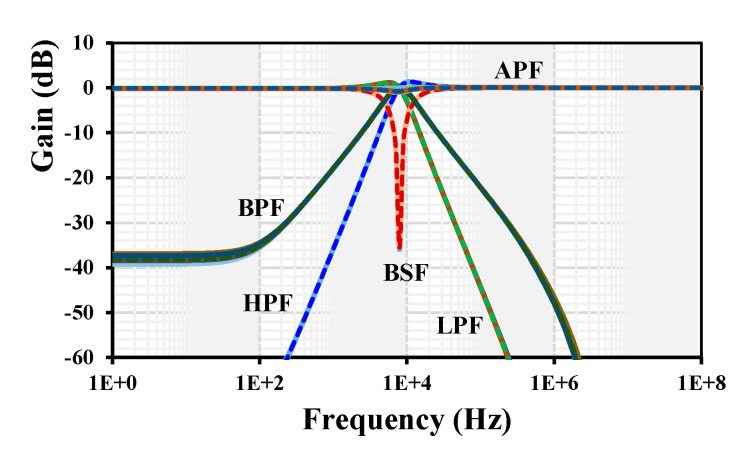
Gain frequency responses of the current-mode filter with PVT corners.

The PVT corners analysis was also used to confirm the robustness of the filter design. The results for the gains frequency responses of the LPF, HPF, BPF, BSF, and APF with PVT are shown in Figure 19. The curves of each filter response overlap, which confirms the robustness of the filter design.

The proposed EDDCC was compared with previous current conveyors in [18,21,23,33,41], as shown in Table 3. Current conveyors using nonconventional techniques, i.e., the bulk-driven CCII [18], bulk-driven DDCC [21], floating-gate FDCCII [23], and current conveyors providing current gain [33,41] have been selected for comparison. Compared with the current conveyors in [18,21,23], the proposed EDDCC offers current gain between z- and x-terminals. Compared with the ECCIIs in [33,41], the proposed EDDCC has much lower power consumption and lower supply voltage. It is worth noting that the bandwidth of the proposed EDDCC is sufficient for many applications like sensors and biomedical systems.

## 5. Experimental Measurements

**Figure 20:**
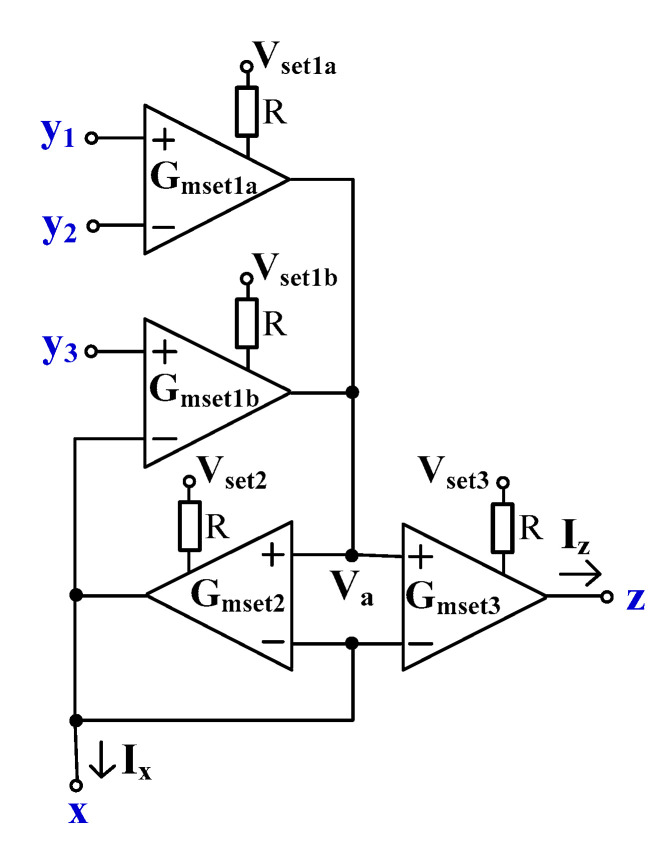
EDDCC using LM13700 devices.

**Figure 21:**
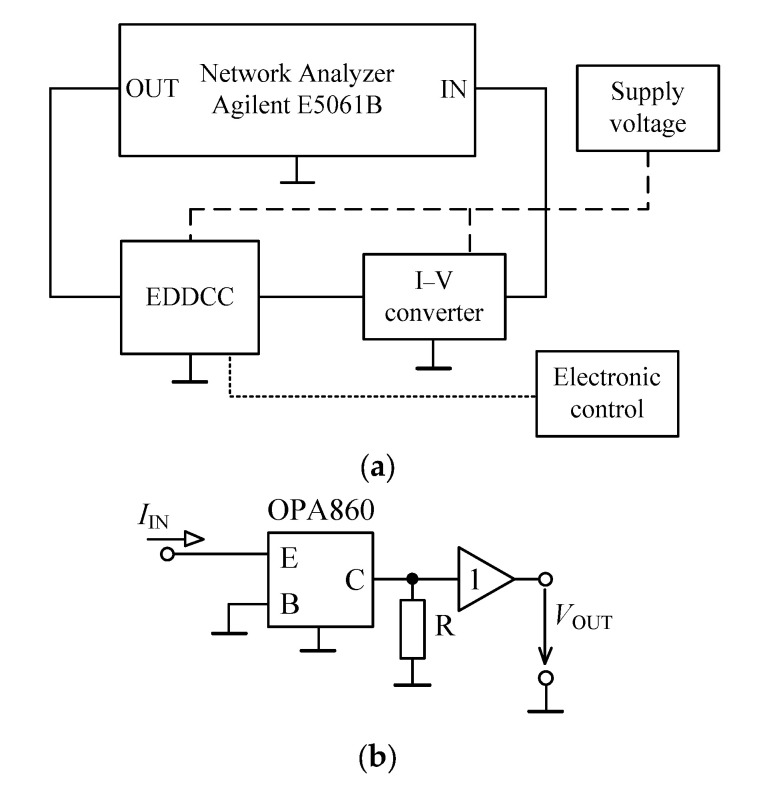
Block diagram of the measurement setup (**a**) and the used I/V converter (**b**).

**Figure 22:**
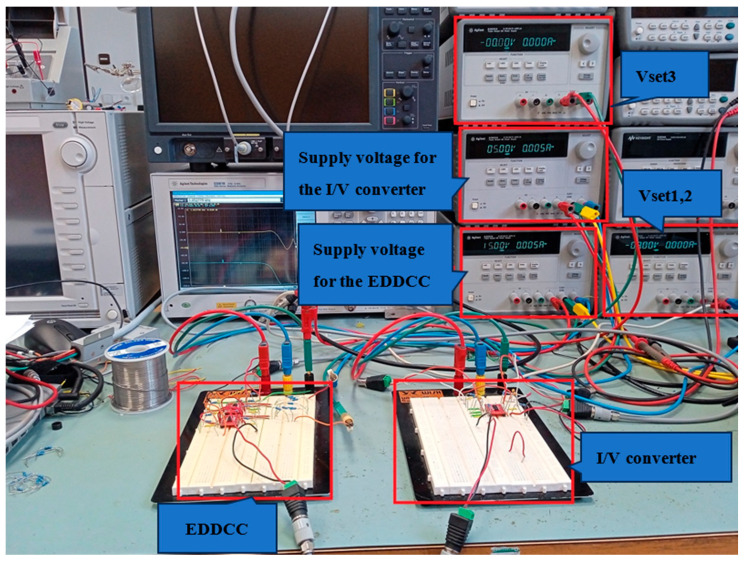
Experimental setup.

The proposed EDDCC was implemented using a commercially available LM13700 device (Texas Instruments, Dallas, TX, USA) [57]. The LM13700 IC uses a ± 15 V supply voltage and its transconductance is controlled by DC current. Since the EDDCC requires an OTA with four inputs (see Figure 4) and the LM13700 device has two inputs, the EDDCC has been implemented using four LM13700 (see Figure 20), where one provides inputs y_1_ and y_2_ and another provides input y_3_ (the positive input of the LM13700 is y_3_, while the negative input is connected to node x). Therefore, G_mset1_ transfer has been divided into G_mset1a_ and G_mset1b_ while these transfers are set to an identical value, thus, G_mset1a_ = G_mset1b_ = G_mset1_. The output currents of these two OTAs are then summed in node V_a_. The control of the transconductances of the LM13700 devices, in this particular implementation, is performed by the control DC voltage V_set_ (the control DC current setting transconductances is controlled by these voltages while the value of the resistor R is kept constant (32 kΩ)). The measurement has been performed using a network analyzer Agilent E5061B, generator Keysight 33500B, and oscilloscope Keysight CX3324A with a current probe CX1101A (Keysight, Santa Rosa, CA, USA). Figure 21a represents a block diagram of the used measurement setup while using the network analyzer. A simple I/V converter (shown in Figure 21b) based on a commercially available OPA860 IC (Texas Instruments) [58] has been used. Its function is as follows: the OPA860 serves as a current follower, the output current is transferred into voltage by the resistor R, and the node with the resistor R is separated from the converter output by a buffer (included in the OPA860 IC) for better impedance properties. The converter uses a supply voltage of ±5 V. A photo of the measuring workplace is depicted in Figure 22.

**Figure 23:**
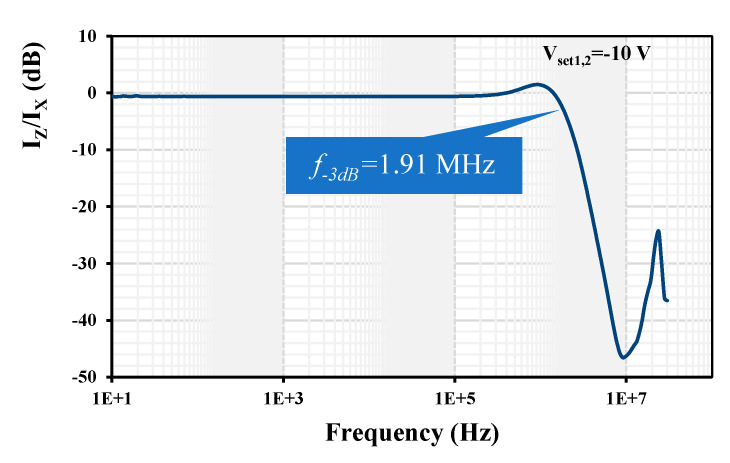
Measured frequency response of the current gain I_z_/I_x_.

The default values for the measurement were selected as follows: R_x_ = 1 kΩ, V_set1_ = V_set2_ = −10 V, and V_set3_ = 0 V. This way, the transconductance of the EDDCC is approximately 1 mS, corresponding to the resistor (1 kΩ) used in the I/V converter for the conversion of the current from the EDDCC back to voltage, which is then fed back to the network analyzer. Note that the resulting voltage transfer corresponds to I_z_/I_x_ transfer when taking the transfer of the I/V converter as a constant. Figure 23 shows the measured current gain I_z_/I_x_ for the default setting. The measurement has been performed in band from 10 Hz up to 30 MHz (the analyzer bandwidth range). The −3 dB bandwidth was measured at 1.91 MHz. The gain is −0.53 dB (note that the resulting gain is given by how accurately the transfer of the EDDCC compensates the transfer of the I/V converter).

**Figure 24:**
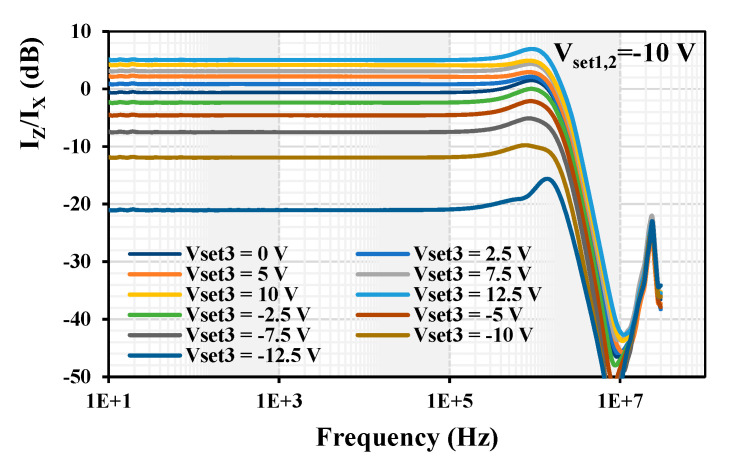
Measured frequency response of the current gain I_z_/I_x_ for different values of V_set3_.

**Figure 25:**
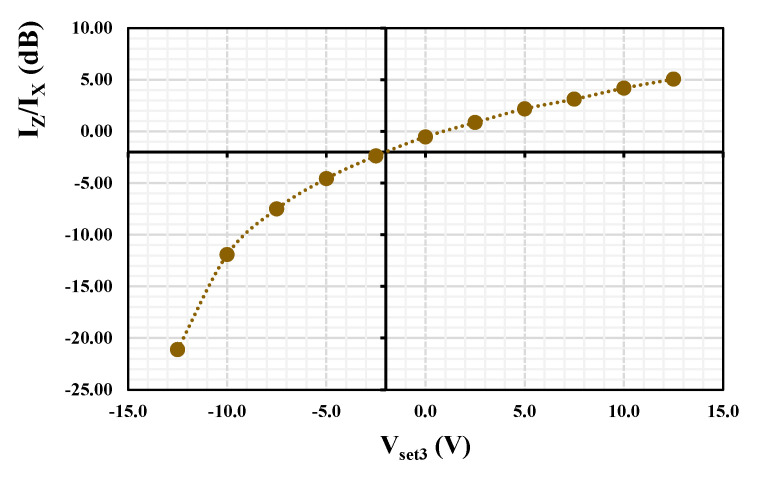
Dependency of current gain I_z_/I_x_ on the control voltage V_set3_.

The possibility to change the current gain I_z_/I_x_ by varying V_set3_ = (−12.5, −10, −7.5, −5, −2.5, 0, 2.5, 5, 7.5, 10, 12.5) V is shown in Figure 24. The obtained current gain was (−21.11, −11.92, −7.50, −4.57, −2.37, −0.53, 0.86, 2.18, 3.11, 4,18, 5.05) dB, respectively. Figure 25 depicts the dependency of the current gain I_z_/I_x_ on the control voltage V_set3_. It shows a logarithmic dependency of the current gain on the control voltage based on this particular implementation.

**Figure 26:**
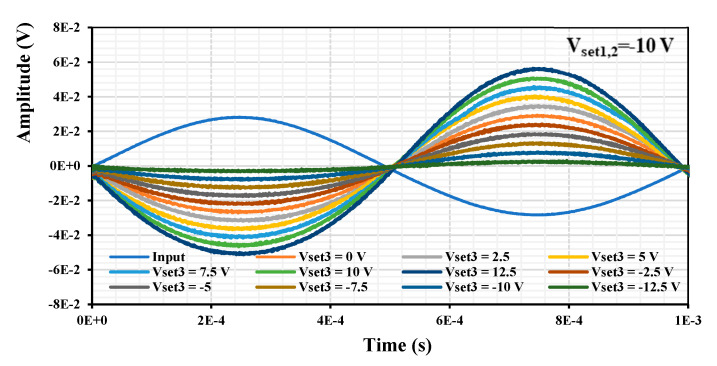
Measured time domain responses for different values of V_set3_.

The measured time domain results of voltage-to-voltage transfer (input of the EDDCC and output of the I/V converter) are presented in Figure 26. The input signal measured at the input of the EDDCC had an amplitude of 57 mV and frequency of 1 kHz. The output signal for V_set3_ = (−12.5, −10, −7.5, −5, −2.5, 0, 2.5, 5, 7.5, 10, 12.5) V is (6, 15, 26, 35, 44, 55, 66, 76, 85, 97, 107) mV, which provides the gain (−19.55, −11.60, −6.85, −4.24, −2.25, −0.31, 1.27, 2.50, 3.47, 4.62, 5.47) dB. There is a 180° of the output in comparison to the input given by the I/V converter (its transfer is inverting). These values correspond well with the values of the current gain I_z_/I_x_ obtained by the analyzer.

## 6. Conclusions

In this paper, a new low-voltage low-power electronically tunable current conveyor has been proposed. Unlike previous current conveyors, the current gain of the proposed current conveyor can be controlled electronically. The proposed current conveyor can work as an electronically tunable DDCC (EDDCC) and an electronically tunable CCII (ECCII). To show the advantages of the current gain of the proposed current conveyors, the V-to-I converter and current-mode universal filter were presented, and the simulation results confirm the functionality of the proposed circuits. The proposed EDDCC uses ±0.5 V power supply, consumes 90 μW of power, and has a ±200 mV DC voltage range, ±10 μA DC current range, and 90 μV voltage offset. The proposed circuit can also offer a 2.81 MHz bandwidth of the voltage gain V_x_/V_y_, a 1.58 MHz bandwidth of the current gain I_z_/I_x_, and a current gain of −9.3 to 14.22 dB when the bias current is varied from 1.25 μA to 40 μA. In addition, the experimental measurements of the EDDCC further support the concept and its functionality.

## Figures and Tables

**Table 1:** Obtaining variant filtering functions of the current-mode universal filter.

Filtering Function		Input	Output	Condition	Gain
LP	Inverting	$I_{1}$	$I_{o1}$	-	$k_{5}$
	Non-Inverting	$I_{2}$	$I_{o2}$	-	$k_{4}$
HP	Inverting	$I_{1}=I_{2}=I_{3}$	$I_{o2}$	$k_{4}=1$	1
BP	Non-inverting	$I_{1}=I_{2}$	$I_{o1}$	-	$k_{5}$
	Non-inverting	$I_{1}$	$I_{o2}$	-	$k_{4}$
BS	Inverting	$I_{1}=I_{3}$	$I_{o2}$	$k_{4}=1$	1
AP	Inverting	$I_{1}=I_{3}$	$I_{o2}$	$k_{4}=2$	1

**Table 2:** Parameters of the components of the MI-OTA.

Transistor	W/L (µm/µm)
M_1_–M_4_, M_13_–M_18_	10/0.5
M_1s_, M_2s_	5/0.5
M_5_–M_12_	20/0.5
M_R_	4/5
C_B_ = 0.5 pF	
V_B1_ = −300 mV, V_B2_ = 200 mV	

**Table 3:** Properties comparison of this work with those of previously published ECCIIs.

Parameters	Unit	This Work	[18]	[21]	[23]	[33]	[41]
		EDDCC	CCII	DDCC	FDCCII	CCII	CCCII
Technique	-	BD	BD	BD	FG	GD	-
Technology	-	0.18 μm CMOS	0.18 μm CMOS	0.18 μm CMOS	0.18 μm CMOS	0.35 μm CMOS	BJT ALA400-CBIC-R
Power supply	V	±0.5	±0.4	±0.3	±0.8	±1.5	±1.5
Power consumption	mW	0.09 (90 µW)	0.064	0.0186	<3	6.6	2.2
Voltage gains:							
V_x_/V_y1_,	-	0.996	1	1	0.94	1	0.99
V_x_/V_y2_,	-	0.995	-	1	-	-	-
V_x_/V_y3_	-	0.996	-	1	-	-	-
Current gain	-	k	1	1	1	k	k
DC voltage range	mV	−200 to 200	−380 to 380	−150 to 150	−1000 to 1000	−500 to 600	−700 to 700
Voltage offset	μV	~90	−0.4 to 0.5	<93	-	-	1.29 to −1.72
DC current range	μA	−10 to 10	−7 to 7	−8 to 8	−300 to 300	−50 to 50	−200 to 200
Current offset	nA	~−2.3	−0.9 to 0.4	<3	-	-	0.0596 to −0.0497
−3 dB bandwidth:		[C_L_ = 0.1 pF]					
V_x_/V_y1_,	MHz	3.16	14	27	-	107	70
I_z_/I_x_	MHz	1.58	13	27	>1000	77	19
Parasitic parameters:							
R_x_/L_x_	Ω/mH	284/18.5	27/860	2.6 k/270	300	46/240	275/0.119
R_y1_/C_y1_	GΩ/fF	42/252	272/117	119/5	-	**∞**/2.7	748 × 10^−3^/491
R_z_/C_z_	MΩ/fF	32.5/52	0.89/40	10.38/0.13	-	73/0.35	814 × 10^−3^/916

Note: V_x_/V_y1_ = V_x_/V_y3_ of CCII, GD = gate driven, BD = bulk driven, FG = floating gate.

## Data Availability

Data are contained within the manuscript.
